# Load-resilient shingled photovoltaic module for field-scale thermoelectric coupling

**DOI:** 10.1038/s41598-026-56895-7

**Published:** 2026-06-13

**Authors:** Kyuhyeon Im, Sungeun Park, Yong Jun Kim, Yonghwan Lee, Kwan Hong Min, Sang Hee Lee, Soo Min Kim, Min Gu Kang, Kyung Taek Jeong, Hae-Seok Lee, Byung Jin Cho, Hee-eun Song, Tae Kyung Lee, Ka-Hyun Kim

**Affiliations:** 1 Photovoltaics Research Department, Korea Institute of Energy Research, Daejeon, 34129 South Korea; 2 Department of Physics, Chungbuk National University, Cheongju, 28644 South Korea; 3 Graduate School of Energy and Environment, Korea University, Seoul, 02841 South Korea; 4 School of Electrical Engineering, Korea Advanced Institute of Science and Technology, Daejeon, 34141 South Korea; 5 Advanced Batteries Research Center, Korea Electronic Technology Institute, Seongnam, 13509 South Korea; 6 AI Convergence R&D Group, R&D Innovation Division, Korea Institute of Ceramic Engineering and Technology, Jinju, 52851 South Korea; 7 Department of Materials Engineering and Convergence Technology, Gyeongsang National University, Jinju, 52828 South Korea

**Keywords:** Hybrid solar conversion, Photovoltaic, Thermoelectric generator, Shingled PV module, Loss analysis, Numerical simulation, Energy science and technology, Engineering

## Abstract

Photovoltaic (PV) solar cells generate waste heat during field operations, which reduces their overall power output. One potential solution for future solar power technology is to integrate solar cells with thermoelectric generators (TEGs) to enable waste heat reclamation, thereby enhancing power output. However, the high TEG resistance (*R*_TEG_) increases the series resistance of these devices, leading to significant power loss. Here, we demonstrated that PV operation at low current and high voltage sufficiently reduces the impact of *R*_TEG_, facilitating field-scale PV–TEG coupling. Furthermore, to achieve low-current, high-voltage operation, a shingled PV module configuration proved effective. This module comprises narrow strip-shaped solar cells connected in series; hence, the current is divided, and the voltage output across the strips is increased. Consequently, lower current and higher voltage than those of an uncut cell of the same size are achieved. Particularly, for a 14-strip shingled module, a load-resilient shingled PV module was realized for a field-scale PV–TEG (170 cm^2^) that delivers 3.27 W with a *P*_loss_ of only 0.043%. This shingled configuration is versatile and can be applied to any solar cell type, including organic, perovskite, and state-of-the-art tandem solar cells. Our study provides potential solutions to the problem of high *R*_TEG_ and new directions for achieving load-resilient PV modules for reliable field-scale PV–TEG coupling.

**Supplementary Information:**

The online version contains supplementary material available at https://doi.org/10.1038/s41598-026-56895-7.

## Introduction

The Sun is a fundamental and inexhaustible source of energy. To address global climate change, expanding the utilization of carbon-free solar energy is essential to power a sustainable future^1^. In order to achieve multi-terawatt scale solar energy use, various studies have focused on effective material consumption^2^, implementing cradle-to-cradle recycling^3^, and developing advanced solar energy harvesting devices^4,5^. The use of photovoltaic (PV) solar cells to convert sunlight into electricity is a carbon-free solution that is more cost-effective than coal or nuclear energy^6^. In 2024, PVs accounted for 6.9% of electricity worldwide, and this proportion is expected to rise^7^. Crystalline silicon (c-Si) single-junction solar cells are the current mainstream solar cells of the industry. Recent progress has pushed the performance of silicon heterojunction solar cells toward fundamental efficiency limits^8^. To further enhance efficiency, specific strategies have been explored, such as developing transparent-conductive-oxide-free front contacts^9^, studies on defect control^10,11^, carrier-selective contacts^12^, and novel material growth techniques^13^. Finally, understanding and mitigating heat generation in these devices remains critical for reliable operation^14^.

However, the performance of these cells is approaching their maximum theoretical efficiency, as they absorb only photons with energies higher than their bandgap and transmit all other lower-energy photons in the solar spectrum^8^. Moreover, in PV cells, excess energy from high-energy photons is dissipated as heat^8,14^. Tandem solar cells, in which two or more subcells with different bandgaps are stacked to capture a broader range of the solar spectrum, are expected to become the mainstream solar cell technology in the future^4,5,15^. However, these tandem cells require complex fabrication and integration processes involving different materials and layer structures, thereby increasing manufacturing costs. This makes tandem solar cells more expensive than single-junction solar cells. Furthermore, to optimize the efficiency of tandem solar cells, the current output of each subcell must be balanced by splitting the solar spectrum, which is achieved based on a simulated solar spectrum corresponding to a solar zenith angle of 48.19° (AM1.5G). Consequently, tandem solar cell configurations, including three-terminal^16^ and two- or four-terminal devices^17^, face inherent operational challenges^18^. Specifically, these multi-junction systems are highly sensitive to variations in atmospheric parameters^19^ as well as variable spectra and ambient temperatures^20^. This environmental sensitivity often causes spectral mismatch in monolithic tandem stacks^21^, which dictates the limiting efficiency for current-constrained two-terminal devices^22^ and fundamentally affects the overall limiting efficiencies in multigap systems^23^. Additionally, waste heat generation is an inherent challenge in all solar cell applications. This waste heat, generated by infrared (IR) irradiation, recombination, and excess energy from high-energy photons during PV operation^8,14^, decreases the overall power outputs of solar cells in both single-junction and tandem configurations.

**Fig. 1:**
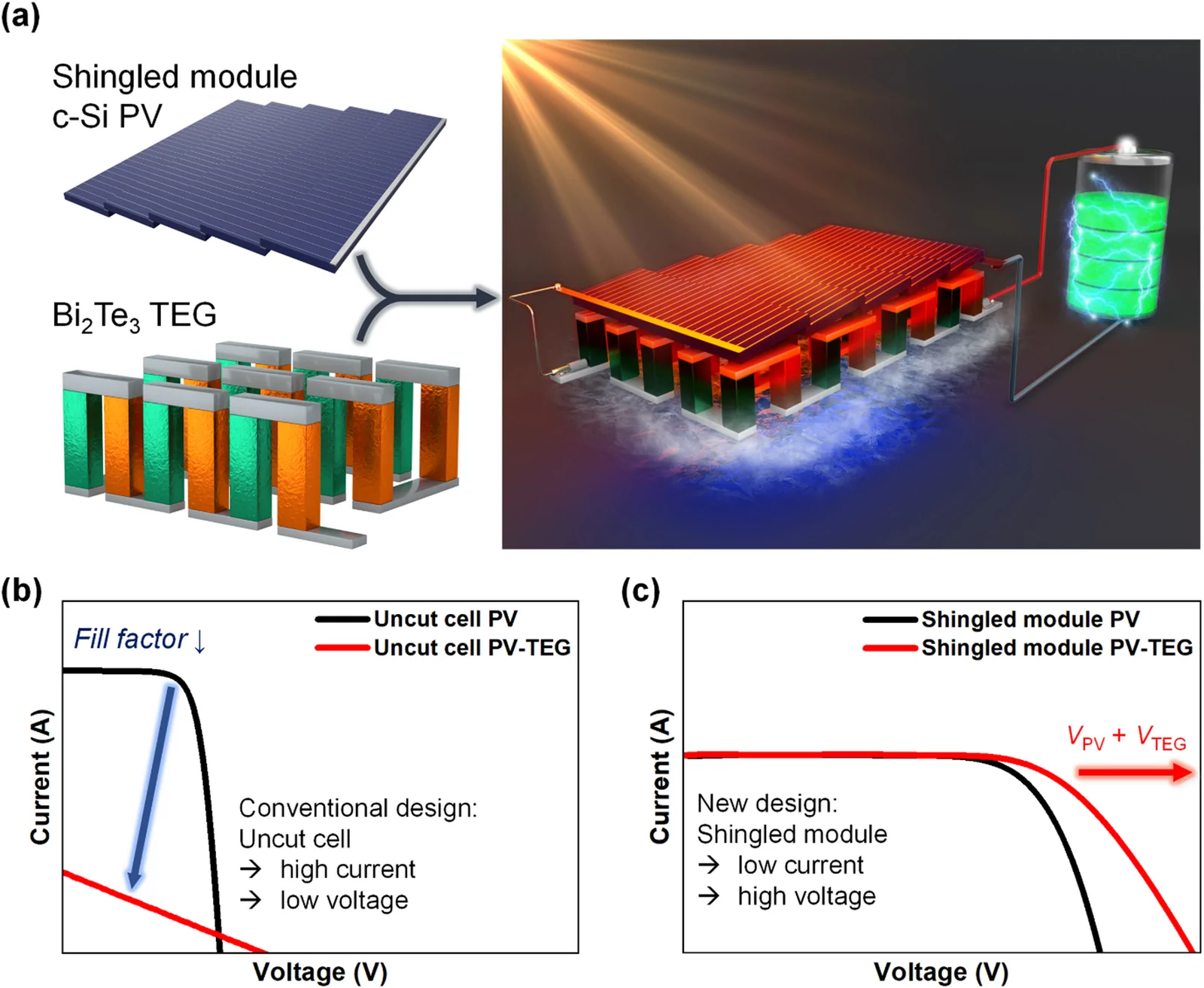
PV–TEG characteristics. (**a**) Schematic illustration of the PV–TEG hybrid coupling. A PV component is connected in series with a TEG that converts the temperature gradient from the PV operation into electricity through the Seebeck effect, thereby providing an additional electromotive force that contributes to the power output. The additional power output from the TEG, combined with that from PV operation, renders the PV–TEG a promising next-generation solar power device. As shown in this work, the PV–TEG can be constructed from commercially available c-Si PV and a Bi_2_Te_3_ TEG connected in series. (**b**) Schematic comparison of *I–V* curves of conventional PV (black) and PV–TEG (red) fabricated with uncut c-Si cells. Owing to the high *R*_TEG_, connecting the TEG and PV in series reduces the PV–TEG *FF*, thereby decreasing the electric power output. This effect is more pronounced in large-area devices, where high-current operation exacerbates *R*_TEG_. Overcoming this challenge is crucial for minimizing the power output loss and transitioning the PV–TEG from the laboratory to the field. (**c**) Schematic comparison of *I–V* curves of PV (black) and PV–TEG (red). The impact of *R*_TEG_ can be minimized by operating the PV at low current and high voltage with a shingled PV module. This shingled module divides the current while increasing the voltage output across the strips, enabling the PV component to operate efficiently at low current and high voltage. *V*_*PV*_ electromotive force from PV, *V*_*TEG*_ thermoelectric electromotive force from TEG.

Hybrid coupling, combining PV cells with thermoelectric generators (TEGs), provides a promising alternative to tandem solar cells^24^. This hybridization strategy has been effectively applied to diverse photovoltaics, including dye-sensitized and organic polymer solar cells^25–27^, as well as perovskite modules and tandem structures^28–30^. Furthermore, the performance of various integrated architectures and monolithic power generators has been extensively investigated^31–33^. Recent efforts have also focused on novel high-performance devices aiming for lossless hybridization^34,35^, along with efficient integrations and numerical thermal analyses^36,37^. Instead of relying on precise spectral splitting across the subcells, the TEG in a PV–TEG hybrid coupling (PV–TEG) reclaims the heat wasted during PV operation by converting the temperature gradient across the device into electricity via the Seebeck effect. This conversion provides an additional electromotive force that contributes to the power output (Fig. 1a). However, despite their excellent prospects, studies on PV–TEGs are currently confined to laboratory-scale testing. Transitioning to practical field-scale PV–TEGs remains challenging because, in a PV–TEG, the TEG resistance (*R*_TEG_) exceeds the PV resistance (*R*_PV_). The high series resistance (*R*_s_, where *R*_PV_ is added to a very high *R*_TEG_) decreases the fill factor (*FF*)^34,35^. The *FF* is the largest rectangular area within the current–voltage (*I*–*V*) curve and determines the maximum power output capability of the device. This reduction in *FF* lowers the electric power output (Fig. 1b). Therefore, minimizing the effects of *R*_TEG_ is key to preventing power output losses and realizing field-scale PV–TEG. However, in the last decade, PV–TEG coupling has been characterized by small area and low power output^25–37^, with *FF* values ranging from 40 to 60%^25–30^. Under such conditions, the *decline* in the *FF* arising from *R*_TEG_ would appear insignificant. Alternatively, PV and TEG components can be operated individually in a four-terminal configuration^30–32^. However, this approach is less preferable because it requires additional wiring and inverters, which increase back-end-of-the-line process and outdoor operation costs. Consequently, the effects of *R*_TEG_ on the power output have been consistently underestimated and/or overlooked.

Here, we demonstrate the significant impact of *R*_TEG_ on the power output of a PV–TEG, and propose a general approach to minimize these effects, thus enabling the development of a load-resilient PV–TEG coupling. Our systematic studies on TEG behavior in the PV–TEG revealed that a high *R*_TEG_ reduces the power output. Moreover, Joule heating arising from current flow across the TEG during operation further increases *R*_TEG_ and exacerbates this loss in power output. However, we discovered that PV operation at low current and high voltage mitigates the impact of *R*_TEG_ (Fig. 1c). Shingled PV modules are ideal for this type of operation because they consist of narrow strips of c-Si solar cells connected in series, which divide the current while increasing the voltage output across each strip. This configuration enables the PV components to collectively deliver a lower current but a higher voltage than an uncut cell of the same size. By integrating a shingled module with a TEG, we realized a field-scale PV–TEG that effectively minimizes power loss under simulated temperature gradient conditions. In addition, we developed a numerical model that can accurately predict the power loss of PV–TEG across various PV parameters. We also studied the slow thermal response of the TEG in the PV–TEG during characterization and addressed such artifacts^38,39^. Remarkably, because these general criteria—i.e., PV operation at low current and high voltage to achieve field-scale PV–TEG—do not depend on the material, this shingled design applies to any solar cell type, including organic, perovskite, and state-of-the-art tandem solar cells. Our study provides potential solutions to the problem of high *R*_TEG_ and new directions towards the realization of a load-resilient PV module for reliable field-scale PV–TEG coupling.

## Methods

### Device fabrication

#### Shingled modules

Commercial passivated emitter and rear contact (PERC) cells composed of c-Si, produced by Shinsung Engineering, were used as the basis for the fabricated shingled modules. These PERC cells were divided into narrow strips using a 1064-nm IR laser for laser scribing, followed by mechanical cleaving. For the shingled modules with three, five, or seven strips, the total designed area was 100 cm^2^; for the 14-strip shingled module, it was 170 cm^2^. The strip dimensions for the shingled modules were as follows: 100 mm × 38.83 mm, 100 mm × 21.70 mm, 100 mm × 16.07 mm, and 85 mm × 16.07 mm for three, five, seven, and 14 strips, respectively. To establish electrical connections, the strips were connected in series using CA 3556HF electrically conductive adhesive, and subsequently hot-pressed and cured at 180 °C for 1 min. The PV tabbing ribbon was soldered at both ends of the shingled modules, which were then encapsulated with a front glass, ethylene-vinyl acetate (EVA) sheet, and polyethylene terephthalate (PET) back sheet. Details of the fabrication process of a shingled module from an uncut cell are provided in the Supplemental Information, along with the raw *I–V* measurements of the shingled PV modules (Supplementary Fig. S1 and S2, respectively).

#### Thermoelectric elements

Commercial thermoelectric (TE) elements were fabricated by Xinrong (China). The *p-* and *n-*type TE materials were Bi_0.5_Sb_1.5_Te_3_ and Bi_2_Te_2.7_Se_0.3_, respectively. The element dimensions were 2 × 2 × 5 mm^3^ (width × depth × height). The Seebeck coefficients for the *p-* and *n-*type elements were found to be 220–230 and 205–215 µV·K^− 1^, respectively; their resistivities were 1000–1050 and 1000–1050 µΩ/cm, respectively; and their thermal conductivities were 1.4–1.6 and 1.3–1.4 mW·cm^− 1^·K^− 1^, respectively. The electrical parameters were measured using a Zem-3 measurement system (ULVAC, Japan) at 22 °C. The material figures of merit were calculated to be 0.777 and 0.748 for the *p-* and *n-*type elements, respectively. The device figures of merit were 0.711 and 0.828, respectively, as measured using the Harman method.

#### TEG fabrication

In this study, 100 cm^2^ TEG arrays were fabricated without a ceramic substrate. The voids between the TE elements within the TEG array were filled with polymeric foam to ensure mechanical robustness. This design facilitated efficient heat transfer to the TEG from heat sources of arbitrary shapes. In most instances, a TEG array with a resistance of 0.88 Ω was employed in the PV–TEG. However, TEG arrays with lower (0.057 Ω) and higher (3.61 Ω) resistances were tested for comparative analysis.

The TEG array with 0.88-Ω resistance employed in this study comprised 77 TE elements arranged in series with a parallel combination of four serial connection lines (77 × 4). The low- (0.057 Ω) and high-resistance (3.61 Ω) TEG arrays comprised 14 × 22 and 154 × 2 TE elements, respectively. In all cases, the total number of TE elements remained consistent at 308, and the total device area was strictly maintained at 100 cm^2^ to ensure a constant heat dissipation area and facilitate meaningful comparisons. The substrate-free TEG array was fabricated by temporarily bonding a patterned Cu film to an adhesive polyimide substrate. For practicality, the polyimide film was temporarily affixed to a glass substrate. Subsequently, a screen printer was used to apply solder paste to the Cu film according to a predetermined pattern. A surface-mounting tool was used to position the TE elements in areas where the solder paste had been deposited. After soldering with a reflow machine, the empty spaces between the TE elements were filled with a polymer to ensure mechanical stability. Finally, the polyimide films were removed to expose the Cu electrodes for electrical connections. Further details can be found in our previous study^27^.

#### Configuration of PV–TEG hybrid coupling

The electrical performance of the PV-TEG hybrid devices was evaluated in two different configurations : two-terminal (2T), where the PV and TEG components are connected in a direct series arrangement requiring only a single pair of external contacts ; and four-terminal (4T), where the PV and TEG are operated as separate electrical circuits with independent contacts to avoid series resistance penalties from the TEG. Throughout this study, the proposed PV–TEG was predominantly configured in the 2T setup, with the 4T configuration reserved specifically for a loss analysis. The flexible design of the TEG array employed in this work, which utilizes thin Cu electrodes and a substrate-free, polymer-filled structure, provided necessary structural adaptability. This adaptability allowed the Cu electrodes to conform securely to the target surface, thereby ensuring good thermal contact without needing a thermal interlayer between the PV and TEG. The 2T configuration involved a simple series connection between the PV and TEG components. In contrast, the 4T configuration incorporated separate electrical contacts for the current inputs and outputs for both the PV and TEG components.

For practical applications, the 2T configuration is preferable owing to its simpler wiring, which results in lower costs during later fabrication stages. In detail, the 4T configuration comprises a top PV and a bottom TEG, and the module is equipped with either a power optimizer or two junction boxes. All top- and bottom-junction boxes must be wired and linked to separate inverters, creating two distinct strings. Consequently, implementing the 4T configuration introduces additional expenses for wiring and inverters during the back-end-of-the-line process and outdoor operation.

### PV–TEG measurements

The *I–V* characteristics of the PV–TEG were measured under two different temperature gradients (Δ*T*), at 0 and 25 °C. For Δ*T* = 0 °C, we aimed to investigate the behavior of the TEG within the PV–TEG and elucidate the operational mechanisms of this hybrid system. The TEG was wrapped with a thermal insulator to isolate it from external temperature effects during the *I–V* measurements. We measured *I–V* while exposing the PV to simulated AM1.5G and 1 sun illumination. To assess the thermal response of the TEG, the integration time (*t*_int_) was varied to 0, 0.5, 1, and 30 s during the measurements. We recorded the temperature changes in the TEG during the *I–V* measurement using a Fortic 340 IR camera and a Fluke 52 II thermometer, such that the temperatures on the hot and cold sides of the TEG could be cross-verified.

Because the performance of the PV–TEG was significantly affected by the temperature gradient, we conducted *I–V* measurements under a constant, simulated Δ*T* = 25 °C. To precisely achieve this Δ*T*, the hot side (PV temperature) was maintained at a constant value of 75 °C using the transparent top heater, and the cold side was maintained at 50 °C using the bottom cooler. This setup was designed to simulate realistic operational conditions, as during prolonged exposure to 1 sun solar radiation, the PV module temperature gradually increases, reaching a saturation point of approximately 80 °C^39^. This temperature contrasts with the standard test conditions (STC) for solar cells, which are conducted at room temperature (25 °C). We designed a custom measurement system equipped with a transparent Cu mesh heater on top and a cooler at the bottom to generate the external temperature gradient required for these experiments. This setup generates an external temperature gradient while simultaneously transmitting simulated solar radiation of AM1.5G and 1 sun through the transparent Cu mesh heater, ensuring the PV module receives the standard irradiance. Further details of this custom-built measurement system can be found in our previous work^39^. Raw *I–V* measurement results for the individual PV, TEG, and PV–TEG are reported in Supplementary Fig. S2 and S3. Further details of the PV–TEG measurements are provided in Supplementary Note S1.

### Characterization of TE elements

For electrical characterization, *p-* and *n-*type TE elements were separated from the TEG array. Hall-effect measurements were performed at room temperature using an HMS-5300 Hall effect measurement system (Ecopia, South Korea). Cu wires were connected to the four corners of the TE elements using conductive Ag paste, thereby forming contacts for the van der Pauw configuration. To accurately determine the electrical properties and reduce experimental uncertainty, the parameters extracted from 20 independent measurement results at different current levels were averaged.

To measure the change in resistance over time, electrical contacts were formed by applying the conductive Ag paste at both ends along the long axis. The TE elements were then connected to a DC power supply, and constant voltage biases of 0.1, 0.35, and 0.7 V were applied to introduce current flow levels of 0.06, 0.2, and 0.4 A, respectively. These voltage levels were chosen to simulate the representative current level for *I–V* measurements on the actual PV–TEG. Under each constant voltage bias, the current flowing across the TE elements was measured every 1 s.

### Numerical model

For the numerical analysis, the PV component was evaluated using an explicit double-diode model (MDDM) solved via the Lambert W-function^40,41^, and the TEG performance was formulated based on the standard governing equations for thermoelectric generators^38^. The detailed formula transformations, comprehensive parameter sets, and step-by-step derivations are provided in the Supplementary Information (Supplementary Note S2). It should be explicitly clarified that the simulated *I–V* curves of the PV-TEG system presented in this study were achieved through mathematical fitting of the experimental data to extract actual operating parameters, such as *R*_TEG_, rather than through pure forward calculation.

The *I–V* curve for this 2T PV–TEG can be expressed as follows:

1 $$\:I\:={I}_{PV}-{I}_{D1}-{I}_{D2}-\frac{V-{V}_{TEG}+I\left({R}_{PV}+{R}_{TEG}\right)}{{R}_{\mathrm{s}\mathrm{h}}}$$

where *I* is the PV–TEG current; *V* is the applied voltage; *V*_TEG_ is the TE electromotive force from the TEG; $\:{I}_{\mathrm{P}\mathrm{V}}$ is the photocurrent generated by the solar cell; $\:{I}_{\mathrm{D}1}$ and $\:{I}_{\mathrm{D}2}$ represent the diffusion and recombination currents, respectively; *R*_PV_ is the series resistance of the PV cell; *R*_TEG_ is the resistance of the TEG; and *R*_sh_ is the parallel resistance^35,40,41^.

We can express *I*_D1_ and *I*_D2_ as follows:

2 $$\:{I}_{\mathrm{D}1}={I}_{01}\left[\mathrm{exp}\left(\frac{V-{V}_{\mathrm{T}\mathrm{E}\mathrm{G}}+I\left({R}_{\mathrm{P}\mathrm{V}}+{R}_{\mathrm{T}\mathrm{E}\mathrm{G}}\right)}{N{n}_{1}{V}_{\mathrm{t}\mathrm{h}}}\right)-1\right]\:\:\:\:\:\:\:\:\:$$

3 $$\:{I}_{\mathrm{D}2}={I}_{02}\left[\mathrm{exp}\left(\frac{V-{V}_{\mathrm{T}\mathrm{E}\mathrm{G}}+I\left({R}_{\mathrm{P}\mathrm{V}}+{R}_{\mathrm{T}\mathrm{E}\mathrm{G}}\right)}{N{n}_{2}{V}_{\mathrm{t}\mathrm{h}}}\right)-1\right]\:\:\:\:\:\:\:\:\:\:\:$$

where *I*_01_ and *I*_02_ are the recombination currents; *N* is the number of strips in the shingled module; *n*_1_ and *n*_2_ are ideality factors; and *V*_th_ is the thermal voltage defined by the Boltzmann constant *k*, operating temperature *T*, and electron charge *q*, and expressed as $\:{V}_{\mathrm{t}\mathrm{h}}=\frac{kT}{q}$. For these two exponential functions to be converted to the Lambert *W* function, it was necessary to combine them into a single exponential function. Therefore, the following process was implemented:

4 $$\:\mathrm{exp}\left(\frac{V-{V}_{\mathrm{T}\mathrm{E}\mathrm{G}}+I\left({R}_{\mathrm{P}\mathrm{V}}+{R}_{\mathrm{T}\mathrm{E}\mathrm{G}}\right)}{N{V}_{\mathrm{t}\mathrm{h}}}\right)={\left(\frac{{I}_{\mathrm{D}1}+{I}_{01}}{{I}_{01}}\right)}^{{n}_{1}}={\left(\frac{{I}_{\mathrm{D}1}+{I}_{02}}{{I}_{02}}\right)}^{{n}_{2}}\:\:\:\:\:\:$$

The expression

5 $$\:{I}_{\mathrm{D}1}+{I}_{01}=\frac{{I}_{01}}{{I}_{02}^{{n}_{2}/{n}_{1}}}{({I}_{\mathrm{D}2}+{I}_{02})}^{{n}_{2}/{n}_{1}}$$

can be further simplified because $\:{I}_{01}$ and $\:{I}_{02}$ are smaller than $\:{I}_{D1}$ and $\:{I}_{D2}$; therefore, $\:{I}_{01}$ and $\:{I}_{02}$ can be neglected. Equation (5) can be simplified to a linear equation and rewritten as follows:

6 $$\:{I}_{\mathrm{D}1}=\kappa\:{I}_{D2}+\tau\:$$

Further, Eq. (1) can be split into Eqs. (7) and (8) by substituting Eq. (6) into the MDDM.

7 $$\:I={I}_{\mathrm{P}\mathrm{V}}-\frac{\kappa\:+1}{\kappa\:}\cdot\:{I}_{01}\left[\mathrm{exp}\left(\frac{\left(V-{V}_{\mathrm{T}\mathrm{E}\mathrm{G}}\right)+I\left({R}_{\mathrm{P}\mathrm{V}}+{R}_{\mathrm{T}\mathrm{E}\mathrm{G}}\right)}{N{n}_{1}{V}_{\mathrm{t}\mathrm{h}}}\right)-1\right]+\frac{\tau\:}{\kappa\:}-\frac{\left(V-{V}_{\mathrm{T}\mathrm{E}\mathrm{G}}\right)+I\left({R}_{\mathrm{P}\mathrm{V}}+{R}_{\mathrm{T}\mathrm{E}\mathrm{G}}\right)}{{R}_{\mathrm{s}\mathrm{h}}}\:$$

8 $$\:I={I}_{\mathrm{P}\mathrm{V}}-\kappa\:+1\cdot\:{I}_{02}\left[\mathrm{exp}\left(\frac{\left(V-{V}_{\mathrm{T}\mathrm{E}\mathrm{G}}\right)+I\left({R}_{\mathrm{P}\mathrm{V}}+{R}_{\mathrm{T}\mathrm{E}\mathrm{G}}\right)}{N{n}_{2}{V}_{\mathrm{t}\mathrm{h}}}\right)-1\right]-\tau\:-\frac{\left(V-{V}_{\mathrm{T}\mathrm{E}\mathrm{G}}\right)+I\left({R}_{\mathrm{P}\mathrm{V}}+{R}_{\mathrm{T}\mathrm{E}\mathrm{G}}\right)}{{R}_{\mathrm{s}\mathrm{h}}}\:$$

Furthermore, the equations comprising an exponential term can be expressed using the Lambert *W* function. Finally, our numerical model for the PV–TEG using MDDM based on the Lambert *W* function can be written as follows:

9 $$\:I\:=\frac{{R}_{\mathrm{s}\mathrm{h}}\left({I}_{\mathrm{P}\mathrm{V}}+{I}_{01}\mathrm{+}{I}_{02}\right)-(V-{V}_{\mathrm{T}\mathrm{E}\mathrm{G}})}{\left({R}_{\mathrm{P}\mathrm{V}}+{R}_{\mathrm{T}\mathrm{E}\mathrm{G}}\mathrm{+}{R}_{\mathrm{s}\mathrm{h}}\right)}-\frac{{NV}_{\mathrm{t}\mathrm{h}}}{{R}_{\mathrm{P}\mathrm{V}}+{R}_{\mathrm{T}\mathrm{E}\mathrm{G}}}\left[\frac{\:\kappa\:}{\kappa\:+1}{n}_{1}\:W{(x}_{1})+\frac{\:1}{\kappa\:+1}{n}_{2}\:W\left({x}_{2}\right)\right]$$

For the loss analysis, the maximum output power (*P*_max_) of the 2T PV–TEG (*P*_max−2T_) was calculated for various combinations of the current at maximum output power (*I*_mp_) and the voltage at the maximum output power (*V*_mp_). The calculated *P*_max−2T_ value was subsequently compared with the *P*_max_ of the 4T PV–TEG (*P*_max−4T_). *P*_max−4T_ was derived by computing the PV-only output power using the MDDM models^40,41^ and adding it to the measured output power of the TEG. Therefore, the power loss (*P*_loss_) can be expressed as follows:

10 $$\:{P}_{\mathrm{l}\mathrm{o}\mathrm{s}\mathrm{s}}\:\left(\%\right)=\left(\frac{{P}_{\mathrm{m}\mathrm{a}\mathrm{x}-4\mathrm{T}}-{P}_{\mathrm{m}\mathrm{a}\mathrm{x}-2\mathrm{T}}}{{P}_{\mathrm{m}\mathrm{a}\mathrm{x}-4\mathrm{T}}}\right)\times\:100$$

Here, the 4T configuration represents an ideal independent operation of the PV and TEG components without the series resistance penalty from *R*_TEG_, whereas the 2T configuration represents the practical series-coupled operation. Therefore, this equation practically quantifies the percentage of generated power that is unavoidably dissipated due to the high *R*_TEG_ when transitioning to a realistic 2T PV-TEG system. Further details of our numerical model are provided in Supplementary Note S2.

## Results and discussion

### Operating PV at low current and high voltage mitigates *R*_TEG_ effects

**Fig. 2:**
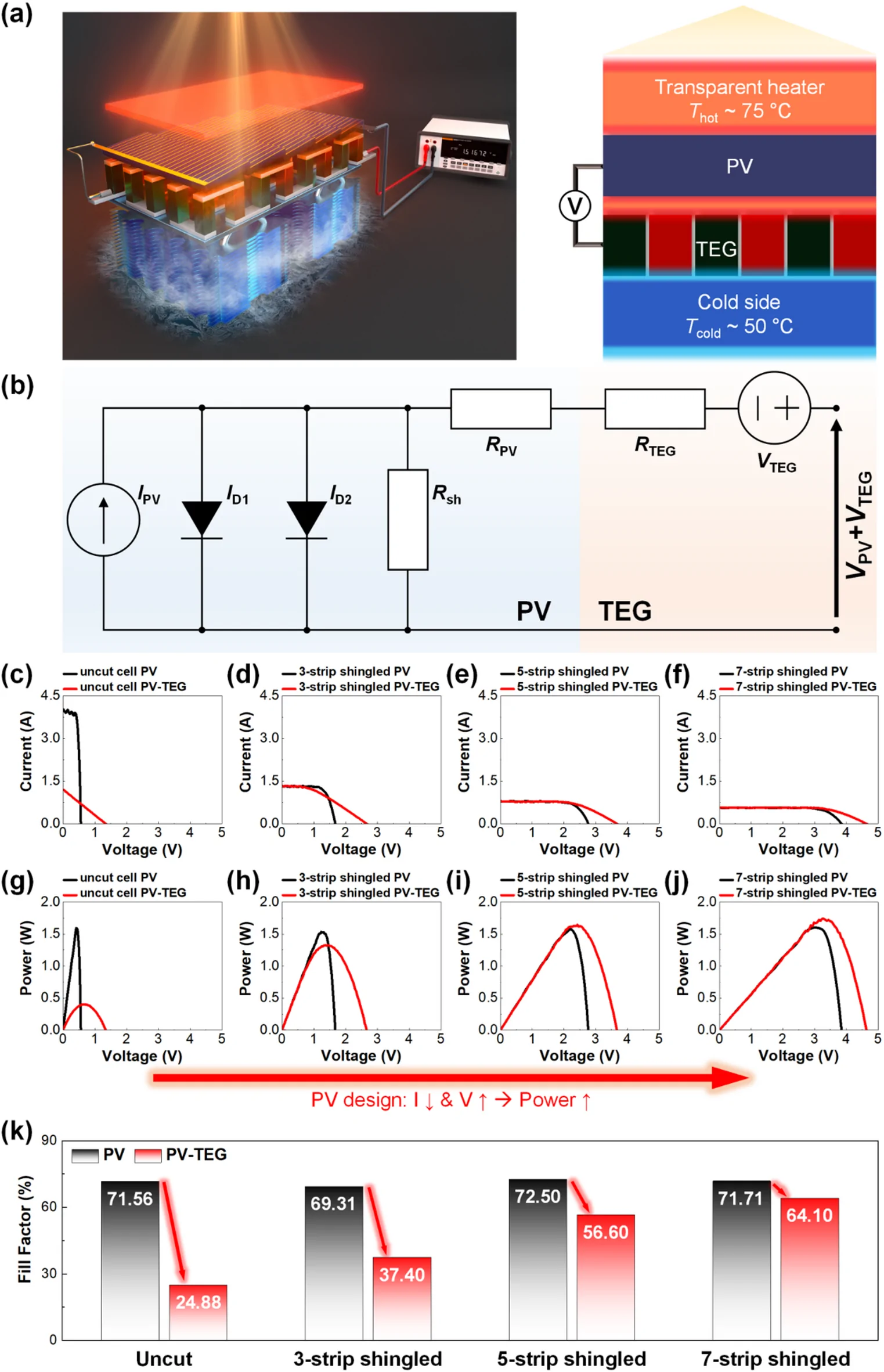
Negligible *P*_loss_ in PV–TEG for PV operating at low current and high voltage to reduce *R*_TEG_ impact. (**a**) Three-dimensional (left) and cross-sectional (right) schematic illustrations of our previously reported custom-built setup for measuring the *I–V* characteristics of PV–TEG^39^. This setup generates an external temperature gradient using a transparent Cu mesh heater while simultaneously transmitting a simulated solar spectrum of AM1.5G and 1 sun. (**b**) Equivalent circuit of our PV–TEG comprising PV and TEG connected in series. Both the thermoelectric electromotive force (*V*_TEG_) from the TEG and the photovoltaic electromotive force (*V*_PV_) from the PV contribute to the PV–TEG power output. However, the *R*_s_ of the device increases significantly because it includes a very high *R*_TEG_. Therefore, minimizing *R*_TEG_ is key to optimizing PV–TEG performance. (**c**–**f**) *I–V* and (**g–j**) *P–V* curves comparing PV (black) and PV–TEG (red) couplings using either uncut cells (**c**,**g**) or shingled modules with three (**d**,**h**), five (**e**,**i**), and seven strips (**f**,**j**). Because shingled modules with more strips operate at lower current and higher voltage than those with fewer strips or uncut cells, they maintain a high *FF* and avoid *P*_loss_. Thus, a PV–TEG with shingled modules is more resilient to the impact of *R*_TEG_. The PV–TEG devices employing five- and seven-strip shingled modules demonstrated power gains. The external temperature gradient was 25 °C, and the PV temperature was approximately 75 °C. (**k**) Comparison of *FF* values between PV (black) and PV–TEG (red) devices using uncut cell and three-, five-, and seven-strip shingled modules. Owing to the high *R*_TEG,_ the PV–TEG devices displayed lower *FF* values than the PV-only devices. The decrease in the *FF* was mitigated when shingled modules were used, suggesting that shingled modules are a key design feature for loss-free PV–TEG.

To explore and identify the conditions that would reduce the impact of *R*_TEG_ on our PV–TEG, we used our previously reported custom-built *I–V* measurement setup (Fig. 2a)^39^. This setup is equipped with a transparent Cu mesh heater on top and a cooler at the bottom, generating an external temperature gradient while simultaneously transmitting simulated solar radiation of AM1.5G and 1 sun. This configuration enabled accurate characterization of our PV–TEG under realistic operating conditions. During the measurements, Δ*T* was set to 25 °C, and the PV temperature (*T*_PV_) was approximately 75 °C^39^. Note that this elevated *T*_PV_ is closer to real-world outdoor operating conditions than the standard test condition of *T*_PV_ = 25 °C^42^.

Based on the equivalent circuit of our PV–TEG (Fig. 2b), the *FF* can be expressed as

11 $$\:FF\approx\:\frac{{V}_{\mathrm{m}\mathrm{p}}{I}_{\mathrm{m}\mathrm{p}}}{{V}_{\mathrm{o}\mathrm{c}}{I}_{\mathrm{s}\mathrm{c}}}-\frac{{I}_{\mathrm{m}\mathrm{p}}^{2}}{{V}_{\mathrm{o}\mathrm{c}}{I}_{\mathrm{s}\mathrm{c}}}\left({R}_{\mathrm{P}\mathrm{V}}+{R}_{\mathrm{T}\mathrm{E}\mathrm{G}}\right)-\frac{{V}_{\mathrm{m}\mathrm{p}}^{2}}{{{V}_{\mathrm{o}\mathrm{c}}{I}_{\mathrm{s}\mathrm{c}}R}_{\mathrm{s}\mathrm{h}}}$$

where *V*_mp_ and *I*_mp_ are the voltage and current at the maximum power point, respectively; *V*_oc_ is the open-circuit voltage; *I*_sc_ is the short-circuit current; and *R*_Sh_ is the shunt resistance of the solar cell ^43^. Because *R*_sh_ is extremely high, its effect can be neglected. Therefore, Eq. (11) can be simplified to

12 $$\:FF\approx\:\frac{{V}_{\mathrm{m}\mathrm{p}}{I}_{\mathrm{m}\mathrm{p}}}{{V}_{\mathrm{o}\mathrm{c}}{I}_{\mathrm{s}\mathrm{c}}}-\frac{{I}_{\mathrm{m}\mathrm{p}}^{2}}{{V}_{\mathrm{o}\mathrm{c}}{I}_{\mathrm{s}\mathrm{c}}}\left({R}_{\mathrm{P}\mathrm{V}}+{R}_{\mathrm{T}\mathrm{E}\mathrm{G}}\right)\approx\:\frac{{V}_{\mathrm{m}\mathrm{p}}{I}_{\mathrm{m}\mathrm{p}}}{{V}_{\mathrm{o}\mathrm{c}}{I}_{\mathrm{s}\mathrm{c}}}\left[1-\frac{{I}_{\mathrm{m}\mathrm{p}}}{{V}_{\mathrm{m}\mathrm{p}}}\left({R}_{\mathrm{P}\mathrm{V}}+{R}_{\mathrm{T}\mathrm{E}\mathrm{G}}\right)\right]$$

From Eq. (12), the *R*_s_ of the PV–TEG comprises *R*_PV_ and *R*_TEG_; therefore, an *R*_TEG_ considerably larger than *R*_PV_ in the PV–TEG decreases the *FF*. The *FF* indicates how closely the *I–V* curve resembles a rectangle, and the area of the rectangle that fits within the *I–V* curve directly corresponds to the power output (*P* = *IV*). A high *R*_s_ causes the *I–V* curve to deviate from a rectangular shape. Consequently, the decrease in *FF* caused by a high *R*_TEG_ decreases the power output of the PV–TEG. Equation (11) also shows that reducing the *I*_mp_-to-*V*_mp_ ratio by operating the solar cell under a low current and high voltage could mitigate the impact of *R*_TEG_. As shingled modules operate at lower current and higher voltage than uncut cells of the same area, they are ideal PV components for PV–TEG.

Overall, to achieve loss-free PV–TEG for field-scale applications, the impact of *R*_TEG_ can be minimized by implementing one of two approaches: reducing *R*_TEG_ or incorporating a PV less susceptible to *R*_TEG_. Although the *R*_TEG_ can be easily reduced in practice by decreasing the number of series connections and/or increasing the number of parallel connections in the TEG array, this approach is undesirable because it increases the operational current and heightens the risk of Joule heating proportional to *I*^*2*^*R.* Further, a TEG array with parallel connections provides a low thermoelectric electromotive force; thus, this configuration would not yield an efficient PV–TEG. Therefore, the best way to attain a high-performance PV–TEG is to use a PV component that is less affected by *R*_TEG_.

Thus, we compared the *I–V* characteristics of PVs and PV–TEGs using uncut cells or shingled modules with three, five, or seven strips. The *I–V* curves (Fig. 2c–f) and the corresponding power–voltage (*P–V*) curves (Fig. 2g–j) show that increasing the number of strips in the shingled module increased *FF* and decreased *P*_loss_. In addition, the PV–TEGs with five- and seven-strip shingled modules displayed power gains (Supplementary Tables S2 and S3). These results demonstrate that operating the PV component at low current and high voltage makes the PV–TEG less susceptible to the influence of *R*_TEG_.

Consistent with previous studies^26,34,35^, because of the high *R*_TEG_, the PV–TEG in this study displayed a significantly lower *FF* than that of the PV-only devices (Fig. 2k; see Supplementary Fig. S2 and S3 for the raw *I–V* measurements for the PV, TEG, and PV–TEG). Importantly, when PVs were coupled with a TEG, a less pronounced decline in *FF* was observed for the shingled PV modules compared to the results for an uncut cell with the same area. This mitigated *decrease in FF* observed for shingled modules suggests a key design feature for a load-resilient PV for field-scale PV–TEG.

### High *R*_TEG_ undermines PV–TEG power output

**Fig. 3:**
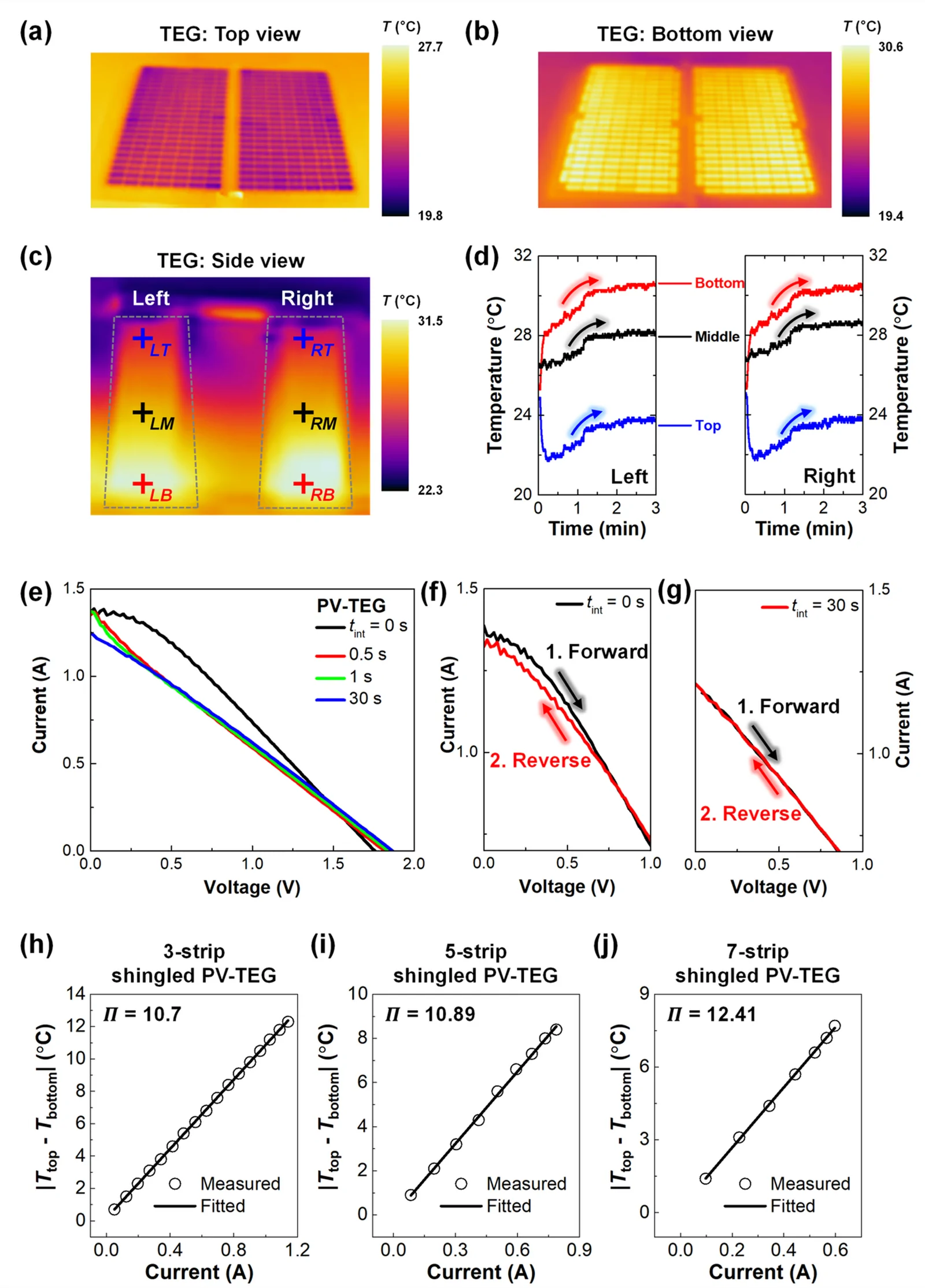
High *R*_TEG_ deteriorates PV–TEG *FF*, and slow Joule heating in TEG further increases *R*_TEG_. IR thermal images showing (**a**) top, (**b**) bottom, and (**c**) side views of TEG elements (assembled with *p-* and *n-*type Bi_2_Te_3_) in PV–TEG during *I–V* measurements. Current flow induces the Peltier effect, cooling the top (LT, RT) and heating the bottom (LB, RB). (**d**) Changes in TEG-element temperatures over time at the top (LT, RT), middle (LM, RM), and bottom (LB, RB) points are marked in (**c**). The Peltier effect occurred within 5 s of the onset of current flow, whereas Joule heating occurred more slowly over 2 min following the initiation of current flow, increasing the overall temperature of the TEG. (**e**) Effects of integration times (*t*_int_) on PV-TEG *I–V* curves. Because of Joule heating, *R*_TEG_ increased slowly during *I–V* measurements, and the TEG did not reach a thermal steady state at low *t*_int_. The *I–V* curves of the PV–TEG exhibited hysteresis at *t*_int_ = 0 s (**f**) but not at *t*_int_ = 30 s (**g**), because Joule heating is a slow process. Therefore, PV–TEG characterization at *t*_int_ = 30 s was more accurate because the TEG reached a thermal steady state. In the hysteresis test, first (black) and second (red) *I–V* sweeps were sequentially performed in the forward and reverse directions. Linear relationship between PV current and temperature gradient generated on TEG top and bottom for (**h**) three-, (**i**) five-, and (**j**) seven-strip shingled modules. Temperature gradients were generated on the top and bottom of the TEG as functions of the PV current, which was measured without external Δ*T*.

To understand how *R*_TEG_ contributes to power loss in the PV–TEG, we examined the *I–V* characteristics of the device with no external Δ*T* in the TEG, where the PV was illuminated with simulated solar radiation of AM1.5G and 1 sun (100 mW/cm^2^). As the current flowed, *R*_TEG_ increased, rendering characterization of the PV–TEG difficult. This is attributed to the TEG’s slow thermal response^38^. With no external temperature gradient (Δ*T* = 0), infrared (IR) thermal imaging during *I–V* measurements revealed that the PV current flow generates a temperature gradient in the TEG via the Peltier effect, cooling the top of the TEG while heating the bottom (Fig. 3a–c). The temperatures of the top and bottom were recorded during *I–V* measurements under illumination, for the three different shingled module configurations (i.e., three, five, and seven strips). The temperature gradient forms rapidly within 5 s of the current flow being initiated (Fig. 3d). Meanwhile, current flow also introduces Joule heating (*I*^2^*R*) to the TEG, albeit considerably more slowly (i.e., over a period of 2 min after the start of current flow). Therefore, current flowing through the TEG introduces a rapid Peltier effect followed by considerably slower Joule heating. The differential thermal response in the TEG indicates that slow Joule heating is the primary reason for the gradual increase in *R*_TEG_ during *I–V* measurements. This slow thermal response significantly impacts the characterization of the PV–TEG. Particularly, the PV–TEG characterization results depend on the integration time (*t*_*int*_), i.e., the duration during which the voltage at each measurement point is sustained. the *I–V* curves of the PV–TEG exhibited steeper slopes at lower *t*_int_ and more moderate slopes at higher *t*_int_ (Fig. 3e). This difference arose because, during a fast voltage sweep (*t*_*int*_ = 0 s), the TEG could not reach a thermal steady state owing to the slow Joule heating, and *R*_TEG_ increased during the *I–V* measurement. In other words, a faster voltage sweep can make the PV–TEG perform better, which is misleading. We also found that the slow thermal response introduced hysteresis into the *I–V* characteristics during a fast voltage sweep (Fig. 3f). The absence of hysteresis at a slow voltage sweep (*t*_*int*_ = 30 s) indicates that the TEG reached a thermal steady state under these conditions (Fig. 3g). Therefore, when measuring the *I–V* characteristics of the proposed PV–TEG, the thermal response of the TEG during operation must be carefully considered (for a detailed discussion of the TEG behavior during *I–V* measurement, see Supplementary Note S1, Supplementary Fig. S4–S6, and Supplementary Table S1).

The temperature gradient at the top and bottom of the TEG exhibited a linear relationship with the PV current (Fig. 3h–j). The slopes of the linear functions for the three-, five-, and seven-strip shingled modules were found to be 10.7, 10.89, and 12.41, respectively. Note that the linear relationship between the generated temperature gradient *|T*_top_ *– T*_bottom_*|* and the PV current comprises the definition of the Peltier coefficient. Considering the charge-carrier motion across the TEG during PV–TEG operation, the rate of heat generation (*Q*) at the contact can be expressed as a linear function of the current:

13 $$\:Q=\pm\:\varPi\:I$$

where $\:\varPi\:$ is the Peltier coefficient, which indicates the amount of heat energy released or absorbed at a junction. A higher Peltier coefficient suggests more efficient TE conversion^44^. Furthermore, the Seebeck and Peltier coefficients are interdependent. Similar to the Seebeck coefficient, the Peltier coefficient $\:\varPi\:$ is defined as the rate of heat generation *Q* with respect to electrical current *I*, as given by

14 $$\:\varPi\:=S\varDelta\:T$$

Here, *S* and ∆*T* are the Seebeck coefficient and external temperature gradient, respectively^45,46^. Considering the slopes of the linear functions, the relationship between the Seebeck and Peltier coefficients suggests that the PV–TEG functions with considerably higher efficiency when a seven-strip shingled module is employed.

Based on these results, we propose a working mechanism for the TEG in the PV–TEG. Owing to the high *R*_TEG_, connecting the TEG to the PV in series decreases *FF*, thus reducing the power output. Through Joule heating, the current flowing in the PV–TEG further increases *R*_TEG_. Given that *R*_TEG_ reduces the power output of the PV–TEG, an increase in *R*_TEG_ via Joule heating would degrade the performance of the PV–TEG. Therefore, our results unequivocally show that the impact of *R*_TEG_ on the PV–TEG is significant and must be minimized.

**Fig. 4:**
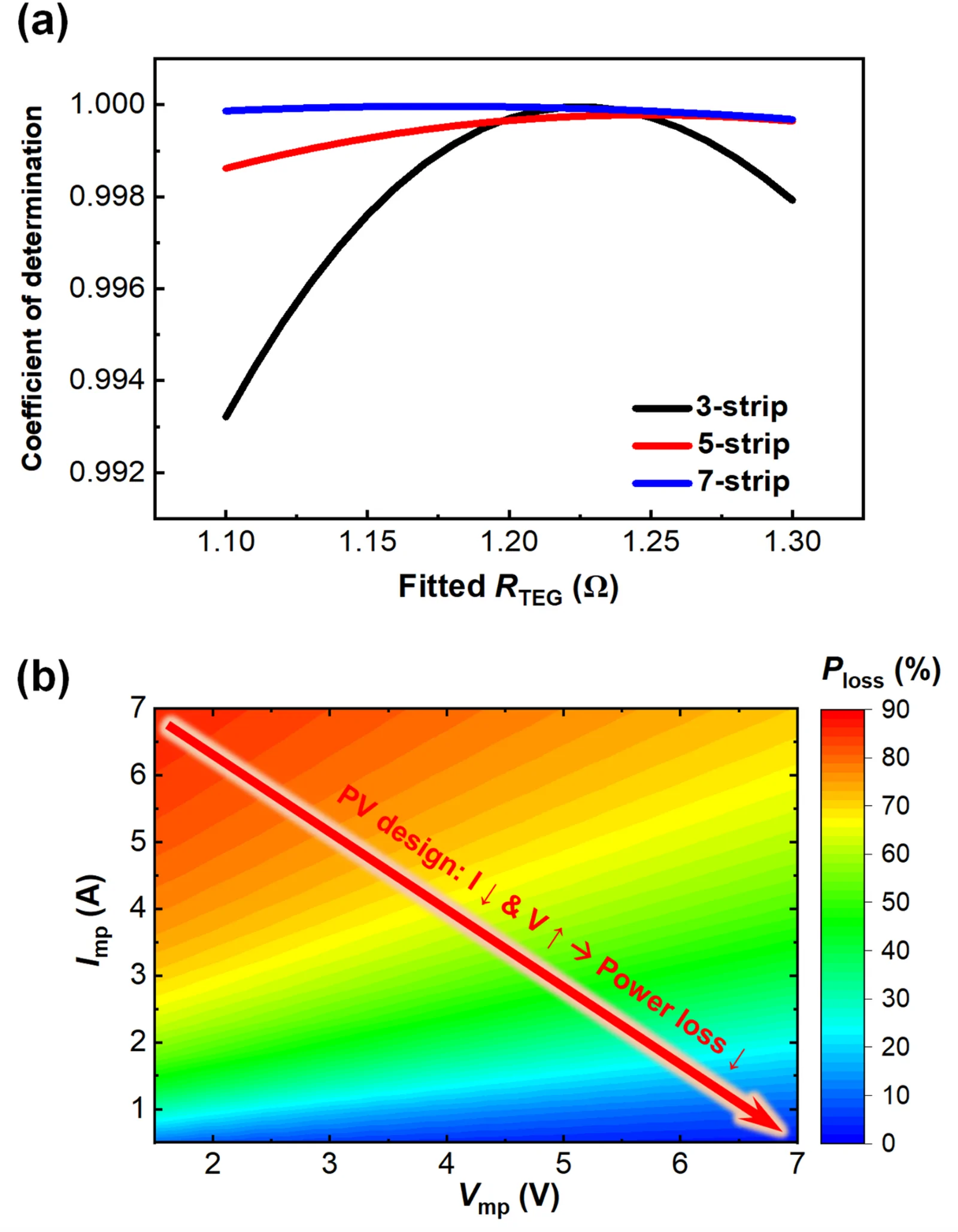
Accurate prediction of PV–TEG power loss by numerical model for various PV parameters. (**a**) Sample fitting results for PV–TEG *R*_TEG_ obtained using a numerical model and considering the maximum coefficient of determination. Applying our numerical model to the measured *I–V* curves of the PV–TEG, we obtained a best fit for *R*_TEG_ at 1.22 Ω instead of the nominal value of 0.88 Ω. This modeling outcome indicates that Joule heating increased *R*_TEG_. (**b**) *P*_loss_ of PV–TEG calculated by our numerical model shown as a function of the PV *I*_mp_ and *V*_mp_. Consistent with the experimental results, our numerical model also predicts that a low *I*_mp_ and high *V*_mp_ minimize the PV–TEG *P*_loss_.

Based on these experimental results, we developed a numerical model to predict the *P*_loss_ of the PV–TEG under various PV parameters (Supplementary Note S2, Supplementary Fig. S7 and S8). Our numerical model for the PV–TEG using MDDM employs the Lambert *W* function to calculate the *I–V* characteristics^40^. Therefore, we simulated *I–V* curves and compared them with the measured *I–V* curves to obtain the maximum coefficient of determination. Loss analysis typically involves fitting the measured *I–V* curve to a numerical model that describes the modeled *I–V* curves of the PV–TEG. The fitting parameters of the numerical model were *I*_01_, *I*_02_, *R*_PV_, *R*_TEG_, and *R*_sh_. By adjusting these parameters during the fitting process, the model was optimized to match the measured data. For example, the effective *R*_TEG_ of the PV–TEG can be deduced from the numerical model (Fig. 4a). Notably, by applying our numerical model to the measured *I–V* curves of the PV–TEG, the best fit for the effective *R*_TEG_ was obtained at 1.22 Ω, instead of the nominal value of 0.88 Ω. This modeling outcome implies an increase in *R*_TEG_ due to Joule heating. Once the model was well fitted to the experimental data, the PV–TEG parameters, such as the maximum output power (*P*_max_), *I*_mp_, *V*_mp_, *I*_sc_, *V*_oc_, and *FF*, could be determined. Our numerical model predicted that the *P*_loss_ of the PV–TEG could be minimized to negligible levels when *I*_mp_ was as low as 0.5 A and *V*_mp_ as high as 7 V (Fig. 4b).

### Field-scale PV–TEG with negligible *P*_loss_

**Fig. 5:**
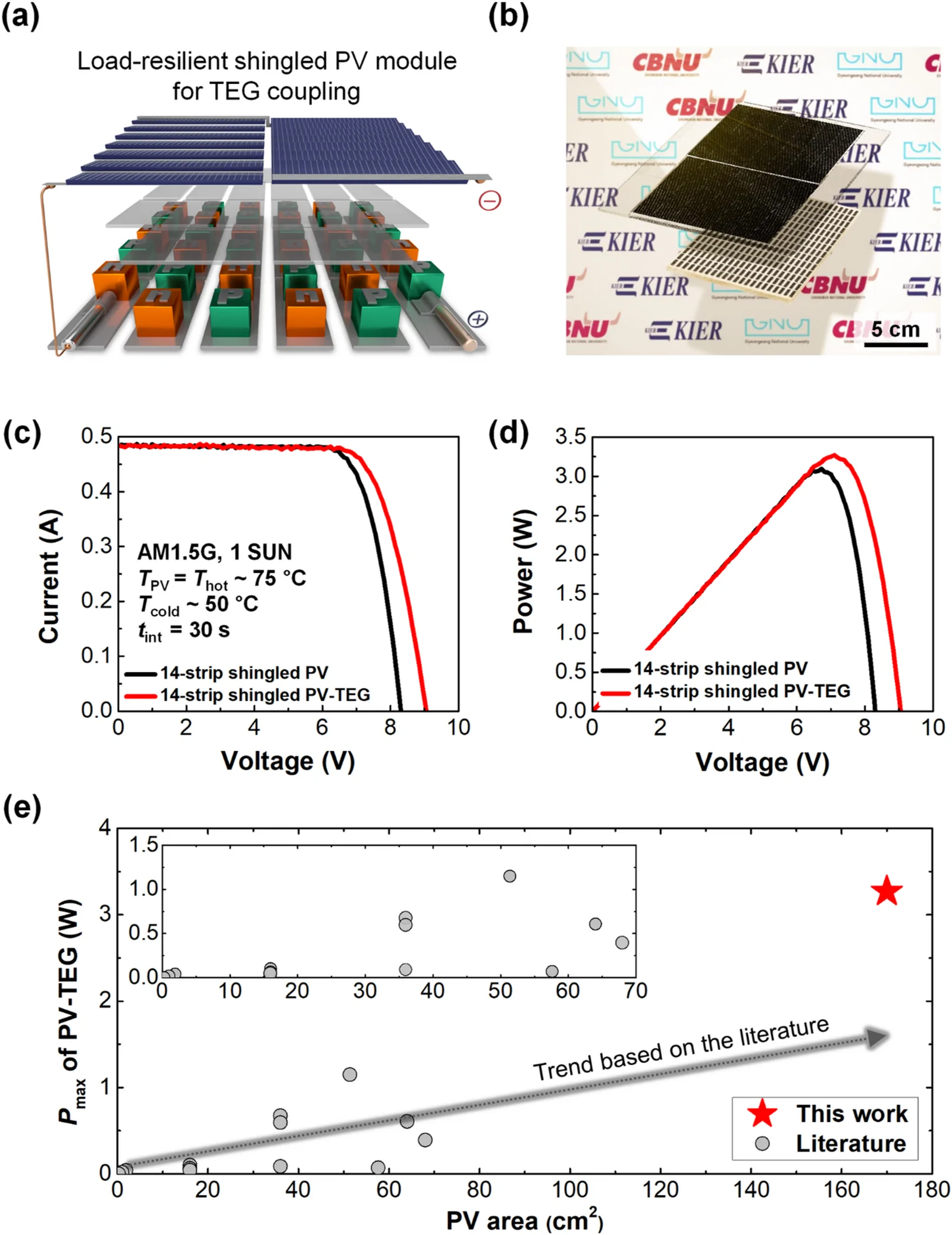
Load-resilient shingled PV module for TEG coupling with negligible *P*_loss_. (**a**) Schematic illustration and (**b**) photograph of our field-scale (170 cm^2^) PV–TEG coupling constructed using a 14-strip shingled module and a TEG. (**c**) *I–V* and (**d**) *P–V* curves of 14-strip shingled modules with PV only (black) and PV–TEG (red), showing power outputs of 3.096 and 3.27 W, respectively. As the TEG power output was 0.1754 W, the PV–TEG *P*_loss_ was only 0.043%. As anticipated from our numerical model, the low-current, high-voltage operation of the 14-strip shingled module successfully suppressed the *P*_loss_ originating from *R*_TEG_. The simulated, external temperature gradient was 25 °C, and the PV temperature was approximately 75 °C. (**e**) Compared to the current state-of-the-art PV–TEG devices reported in the literature^25–37^(gray circles), our PV–TEG (red star) displays the highest power output (3.27 vs. 1.15 W) over the largest area (170 vs. 68 cm^2^). Inset: magnified view of a small-area, low-power region occupied by the best-performing PV–TEG devices reported previously.

Encouraged by the experimental results and predictions of our numerical model, we examined a large-area (170 cm^2^) PV–TEG coupling using a 14-strip shingled module with a low *I*_mp_ of 0.5 A and a high *V*_mp_ of 7 V (Fig. 5a,b, Supplementary Table S4). Under a simulated condition of Δ*T* = 25 °C, our PV–TEG coupled device generated 3.27 W (Fig. 5c,d) and exhibited a *P*_loss_ of only 0.043%, as determined by the measured power outputs of each component in the PV–TEG: 3.096 W for the 14-strip shingled module and 0.1754 W for the TEG. Notably, the total power output of our PV–TEG far exceeds that of the best-performing devices reported in the literature, which currently stands at 1.15 W (Fig. 5e and Supplementary Table S5)^33^. The shingled module, which divides the current while increasing the voltage across multiple strips, effectively suppressed the effect of *R*_TEG_ to achieve such a low *P*_loss_ and enabled our PV–TEG to maintain a low current and high voltage over a much larger area of 170 cm^2^ than the largest area reported thus far in the literature, i.e., 68 cm^2^ (Fig. 5e and Supplementary Table S5)^32^. These results indicate that the impact of *R*_TEG_ can be minimized to achieve field-scale PV–TEG coupling with negligible *P*_loss_, and that our numerical model accurately predicts the *P*_loss_ of the PV–TEG. However, although we successfully demonstrated a load-resilient shingled PV module for a field-scale PV–TEG, this work was limited to a laboratory-based proof-of-concept demonstration, with no actual thermal couplings for realistic devices. Additionally, no outdoor operational tests were performed. Therefore, future research should focus on evaluating the operational reliability of PV–TEG through comprehensive outdoor testing under real-world conditions.

## Conclusions

Our systematic studies showed that *R*_TEG_ substantially affects the performance of a PV–TEG. Specifically, a high *R*_TEG_ decreases the *FF* of the PV–TEG, inducing a loss in the electric power output. We showed that current flowing through the TEG during operation introduces Joule heating, further elevating *R*_TEG_. This heightened *R*_TEG_ contributes further to the loss in power output. Through numerical simulations and experiments, we demonstrated that PV operation at low current and high voltage mitigates the impact of *R*_TEG_. Using a 14-strip shingled module, which divides the current while increasing the voltage across multiple strips, we realized a load-resilient shingled PV module for a field-scale PV–TEG (170 cm^2^) that delivers 3.27 W with a *P*_loss_ of only 0.043%. The scale and performance of our PV–TEG represent significant advances over the largest (68 cm^2^) and best-performing (1.15 W) devices reported thus far in the literature. Unlike tandem solar cells, which require complex monolithic integration and sophisticated spectral splitting, our PV–TEG involves only a straightforward connection of commercially available PV and TEG components, with no front-end-of-the-line fabrication being necessary.

In the future, our load-resilient PV–TEG coupled device should be tested for feasibility with optimized thermal couplings between the PV/TEG and TEG/heat sink interfaces, possibly in outdoor operational tests. Finally, because the general criteria of low-current and high-voltage operation reported here are independent of the material, the shingled PV module used in our PV–TEG can, in principle, be implemented with any type of solar cell, including (but not limited to) organic and perovskite solar cells, as well as state-of-the-art tandem solar cells. Materials with larger bandgaps tend to display lower current and higher voltage than c-Si. Consequently, their application as PV components in the shingled design employed herein could further improve the power output of the resultant PV–TEG.

## Supplementary Information

Below is the link to the electronic supplementary material.


Supplementary Material 1


## Data Availability

The data that support the results of this study are available from the corresponding authors, Hee-eun Song and Ka-Hyun Kim, upon reasonable request.
