# Supplementary material for: Load-resilient shingled photovoltaic module for field-scale thermoelectric coupling
Source: Sci Rep. 2026 Jun 13;16:27041. doi: 10.1038/s41598-026-56895-7 (PMC13522502; doi:10.1038/s41598-026-56895-7)
Supplement: Supplementary file 1 [file 41598_2026_56895_MOESM1_ESM.docx]

**Supplementary Information**

**Load-resilient shingled photovoltaic module for field-scale thermoelectric coupling**

Kyuhyeon Im^†^, Sungeun Park^†^, Yong Jun Kim, Yonghwan Lee, Kwan Hong Min, Sang Hee Lee, Soo Min Kim, Min Gu Kang, Kyung Taek Jeong, Hae-Seok Lee, Byung Jin Cho, Hee-eun Song*, Tae Kyung Lee*, and Ka-Hyun Kim*

^†^ These authors contributed equally to this work.

^*^ Corresponding authors. Email: [hsong@kier.re.kr](mailto:hsong@kier.re.kr) (Hee-eun Song); [tklee8865@gnu.ac.kr](mailto:tklee8865@gnu.ac.kr) (Tae Kyung Lee); [kahyunkim@chungbuk.ac.kr](mailto:kahyunkim@chungbuk.ac.kr) (Ka-Hyun Kim).

**Supplementary Fig. S1.** Schematic illustration of the shingled-module fabrication process using an uncut c-Si cell. The fabricated shingled module was based on commercial PERC cells produced by Shinsung Engineering. These PERC cells were divided into narrow strips using a 1064-nm IR laser for laser scribing, followed by mechanical cleaving. To establish electrical connections, the strips were connected in series using CA 3556HF electrically conductive adhesive, and subsequently hot-pressed and cured at 180 °C for 1 min. The total areas of the shingled modules with three, five, or seven strips were 100 cm^2^; that of the 14-strip shingled module was 170 cm^2^. After the PV-tabbing ribbon was soldered at both ends of the shingled modules, the modules were encapsulated using front glass, an EVA sheet, and a PET back sheet.

**Supplementary Fig. S2.** (**a**) *I–V* curves and (**b**) photographs of uncut c-Si solar cell and three-, five-, and seven-strip shingled modules. Current reductions and voltage increases were observed across the various strips of the shingled modules compared to those of an uncut cell with identical dimensions. This characteristic is attributed to the series connection of the narrow, strip-shaped solar cells within the module. This connection effectively divides the overall current while simultaneously multiplying the voltage output along the strips. Consequently, the low-current and high-voltage operation of the shingled module effectively mitigates the severe power loss originating from the high *R*_TEG_ when coupled with a TEG. This advantageous performance is a key outcome of the innovative design, aligning with our overarching goal of optimizing PV technology for enhanced energy generation. (**c**) *I–V* curve and (**d**) photograph of 0.88-Ω, 77 × 4 TEG array used in this work. Notably, *I–V* measurements were performed for the TEG under various Δ*T* from 0 to 25 °C.

**Supplementary****Fig. S3.** Comparison of *I–V* curves of PV and 2T PV–TEG for different PV configurations: uncut cell and three-, five-, and seven-strip shingled modules. Notably, the *I–V* curves of the PV–TEG were recorded with no external temperature gradient in the TEG (*t*_int_ = 30 s, Δ*T* = 0 °C, *T*_PV_ ~ 25 °C, AM1.5G, and 1 sun). These results yielded two notable findings. First, compared with the PV device, the PV–TEG exhibited significantly lower *FF*, attributable to *R*_TEG_. The lower *FF* in the PV–TEG, induced by *R*_TEG_, is a consistent problem impacting the overall performance of PV–TEG systems. Second, the decreased *FF* in PV–TEG was notably mitigated using shingled modules as opposed to an uncut cell with an equivalent area. This observation underscores a key advantage of using shingled modules in the PV–TEG configuration and suggests a key strategy for enhancing the overall efficiency of PV–TEG.

**Supplementary****Fig. S4.** Current and resistance time evolutions for individual (left) *p-*type and (right) *n-*type TE elements. When subjected to constant voltage, the TE-element resistance gradually increased over time.

**Supplementary****Fig. S5.** Hysteresis in *I–V* curves of 2T PV–TEG with *t*_int_ set to (**a**) 0 and (**b**) 30 s. The first and second *I–V* sweeps were performed in the forward and reverse directions, respectively. When *t*_int_ was set to 0 s, a prominent hysteresis occurred between these two sweeps. However, when the *t*_int_ was set to 30 s, this hysteresis disappeared, and the forward sweep closely mirrored the subsequent reverse sweep. The thermal response of the TEG manifested as a slow process, as shown in Supplementary Fig. S4, and for *t*_int_ = 0 s, the internal temperature of the TEG increased during the *I–V* measurement. Consequently, this temperature rise increased *R*_TEG_. In contrast, when the *t*_int_ was set to 30 s, the internal temperature of the TEG reached a steady state during the *I–V* measurement. This steady-state condition corresponded to a stabilized internal temperature of the TEG, which yielded more consistent and predictable behavior. Consequently, *R*_TEG_ remained constant under this setting. The observed differences underscore the significant impact of the integration time on the thermal dynamics and electrical characteristics of the TEG. This finding emphasizes the importance of choosing an appropriate *t*_int_ to optimize the measurement conditions.

**Supplementary****Fig. S6.** Characterization of 2T PV–TEG with different TEG resistances (*R*_TEG_). *I–V* curves of TEG with (a) low and (b) high *R*_TEG_ of 0.057 and 3.61 Ω, respectively. *I–V* curves of a seven-strip shingled module and PV–TEG in cases of (c) low and high *R*_TEG_ of 0.057 and 3.61 Ω, respectively. The *I–V* curves were measured without Δ*T,* and *t_int_* was set to 30 s.

**Supplementary****Fig. S7.** Measured and modeled *I–V* curves of (left) PV only and (right) 2T PV–TEG in various PV configurations, including uncut cell, three-, five-, and seven-strip shingled modules, as revealed by the *I–V* measurement setup and numerical model developed in this work.

**Supplementary****Fig. S8.** Contour plots of calculated maximum output power (*P*_max_) for (top) 2T PV–TEG and (bottom) 4T PV–TEG, as predicted by numerical model, showing relationship between *I*_mp_ and *V*_mp_ of hybrid devices. The difference between the two *P*_max_ values indicates the power loss presented in Fig. 4(b).

**Supplementary Table S1.** Hall effect measurements of individual *p-* and *n-*type TE elements. Notably, the negative sign of the carrier concentration indicates that the material is *n-*type.

|  | **Resistivity (Ω∙cm)** |  | **Carrier concentration (#/cm^3^)** | **Mobility (cm^2^/V∙s)** |
| --- | --- | --- | --- | --- |
| *p*-type | 5.24 × 10^–2^ |  | 2.01 × 10^19^ | 59.2 |
| *n*-type | 2.01 × 10^–3^ |  | –1.80 × 10^19^ | 279 |

**Supplementary Table S2.** PV parameters from the results presented in Fig. 2 for PV components only.

| **Parameters** | **Uncut** | **Three strips** | **Five strips** | **Seven strips** |
| --- | --- | --- | --- | --- |
| *P*_max_ (W) | 1.594 | 1.526 | 1.569 | 1.599 |
| *V*_oc_ (V) | 0.562 | 1.674 | 2.767 | 3.846 |
| *J*_sc_ (mA/cm^2^) | 42.07 | 13.79 | 8.183 | 5.881 |
| *FF* (%) | 71.56 | 69.31 | 72.50 | 73.99 |
| *Efficiency* (%) | 16.92 | 16.00 | 16.42 | 16.73 |
| Measurement conditions: *t*_int_ = 30 s, △*T* = 0 °C, T_PV_ ~75 °C, AM1.5G, and 1 sun.  *J*_sc_ was calculated using the active surface area of the PV (shading loss ~5%). | | | | |

**Supplementary Table S3.** PV parameters from results presented in Fig. 2 for 2T PV–TEG coupled device

| **Parameters** | **Uncut** | **Three strips** | **Five strips** | **Seven strips** |
| --- | --- | --- | --- | --- |
| *P*_max_ (W) | 0.402 | 1.325 | 1.631 | 1.731 |
| *V*_oc_ (V) | 1.345 | 2.661 | 3.670 | 4.631 |
| *J*_sc_ (mA/cm^2^) | 12.77 | 13.96 | 8.225 | 5.860 |
| *FF* (%) | 24.88 | 37.40 | 56.60 | 66.68 |
| *Efficiency* (%) | 4.267 | 13.90 | 17.07 | 18.11 |
| Measurement conditions: *t*_int_ = 30 s, △*T* = 25 °C, T_PV_ ~75 °C, AM1.5G, and 1 sun.  *J*_sc_ was calculated using the active surface area of the PV (shading loss ~5%). | | | | |

**Supplementary Table S4.** PV parameters of 170-cm^2^ large-area PV–TEG coupled device using 14-strip shingled module results presented in Fig. 5.

| **Parameters** | **14-strip shingled module PV** | **TEG** | **PV–TEG** |
| --- | --- | --- | --- |
| *P*_max_ (W) | 3.096 | 0.1754 | 3.270 |
| *V*_oc_ (V) | 8.303 | 0.765 | 9.050 |
| *J*_sc_ (mA/cm^2^) | 2.973 | 9.95 | 2.979 |
| *FF* (%) | 77.19 | 23.06 | 74.66 |
| *Efficiency* (%) | 19.06 |  | 20.13 |
| Measurement conditions: *t*_int_ = 30 s, △*T* = 25 °C, T_PV_ ~75 °C, AM1.5G, and 1 sun.  *J*_sc_ was calculated using the active surface area of the PV (shading loss ~5%). | | | |

**Supplementary Table S5.** Comparison of various PV–TEGs presented in this work and in the literature

| **References in the main manuscript** | | **Configuration** | **PV area (cm^2^)** | ***P*_max_ (W) of PV–TEG** |
| --- | --- | --- | --- | --- |
| This work | | 2T | 170 | 3.27 |
|  |  | 4T | 170 | 3.2714 |
| [25] | Chen *et al. Energy & Environmental Science* (2012) 5, 6294–6298 | 2T | 0.25 | 0.0018 |
| [26] | Zhang *et al. The Journal of Physical Chemistry C* (2013) 117, 24685–24691 | 2T | 0.12 | 0.0014 |
| [27] | Kim *et al. Nano Energy* (2022) 93, 106775 | 2T | 57.6 | 0.0715 |
| [28] | Liu *et al. Nano Energy* (2017) 38, 457–466 | 2T | 1.875 | 0.0416 |
| [29] | Fu *et al. Nano Energy* (2019) 65, 104009 | 2T | 16 | 0.103 |
| [30] | Guo *et al. Journal of Power Sources* (2010) 195, 7684–7690 | 2T | 16 | 0.0426 |
|  |  | 4T | 16 | 0.0442 |
| [31] | Cotfas *et al. Materials Science and Engineering* (2016) 133, 012037 | 4T | 36 | 0.6748 |
|  |  | 4T | 36 | 0.5951 |
|  |  | 4T | 36 | 0.0884 |
| [32] | Deng *et al. Solar Energy* (2013) 88, 182–191 | 4T | 68 | 0.393 |
| [33] | Kim *et al. ACS Applied Energy Materials* (2019) 2, 2381–2386 | 2T | 51.35 | 1.15 |
| [34] | Wang *et al. Energy & Environmental Science* (2011) 4, 3676–3679 | 2T | 0.0625 | 0.0009 |
| [35] | Park *et al. Scientific Reports* (2013) 3, 2123 | 2T | 16 | 0.0652 |
| [36] | Xu *et al. Advanced Energy Materials* (2018) 8, 1702937 | 2T | 1 | 0.0203 |
| [37] | Darkwa *et al. Applied Energy* (2019) 248, 688–701 | 2T | 64 | 0.608 |

Supplementary Notes

**Supplementary Note S1: Thermal response of TEG: increases in resistance and *I–V* hysteresis during current flow**

This Supplementary Note discusses the thermal response of the TEG component during *I–V* measurements, as shown in Supplementary Fig. S4–S6 and Table S1. As discussed in the main text, the TEG resistance (*R*_TEG_) decreases the output power of the PV–TEG. Therefore, the impact of *R*_TEG_ on the PV–TEG is significant and must be minimized.

During current flow, *R*_TEG_ increases, making PV-TEG characterization more difficult. Supplementary Fig. S4 shows the time evolution of the current and resistance behaviors of individual *p-* and *n-*type Bi_2_Te_3_ TE elements. Under a constant bias voltage, the current in the TE elements decreased over time. When the current flowed through individual TE elements under a constant bias voltage, it first rapidly decreased within the first 30–60 s and then stabilized. This result was attributed to the change in the resistance of Bi_2_Te_3_ over time. In other words, the resistance of the TE elements increased for approximately 30 s following current-flow initiation. The TE element, Bi_2_Te_3_, is a narrow-gap semiconductor with an indirect bandgap of 160 meV at 300 K [S2,S3]. The Hall effect measurement results reported in Supplementary Table S1 indicate that both the *p*- and *n*-type TE elements in the TEG employed in this study were highly doped. High doping concentrations in narrow-gap semiconductors yield degenerate semiconductors [S4], which exhibit metallic conduction characteristics, where the resistance increases with temperature [S5].

Another interesting point is that *I–V* measurements of the TEG were significantly influenced by the integration time (*t*_int_). In the context of *I–V* measurements, the *t*_int_ and delay time (*t*_delay_) are related; however, different parameters apply to certain measurement instruments, such as semiconductor parameter analyzers and other electrical characterization tools. In this case, *t*_int_ refers to the duration during which the measurement instrument collects data or averages readings. Furthermore, *t*_int_ is generally used to achieve enhanced measurement accuracy through reduction of noise and fluctuations in the measured data, as it allows the measurement to dwell at each voltage point for a longer period. For the TEG measurements performed in this study, longer *t*_int_ yielded a sustained current flow, which caused more significant Joule heating. Notably, this sustained current flow accurately represented realistic operating conditions. Therefore, setting a longer *t*_int_ for TEG measurements is more realistic, even if the *I–V* curves appear to be poorer. In contrast, *t*_delay_ is the time interval between measurement initiation and the time at which the instrument begins collecting data. In other words, this is the time delay before the actual measurement process begins. Some devices may exhibit transients or changes in their electrical behavior when a voltage or current is applied, and *t*_delay_ ensures that measurements are recorded under stable conditions. In the case of the TEG measurements, the device remained idle during the *t*_delay_ between measurement points. Setting a longer *t*_delay_ would have decreased the influence of the TEG thermal response, as suggested in the literature [S6].

Supplementary Fig. S5 compares the *I–V* measurements with different voltage sweep directions in the forward and reverse sweeps. Significant hysteresis was observed when *t*_int_ was set to 0 s; however, no significant difference was observed when *t*_int_ was set to 30 s. The first *I–V* sweep was conducted in the forward direction and was followed by a second *I–V* sweep in the reverse direction. The slope of the *I–V* curve obtained using a fast sweep (*t*_int_ = 0 s) was steeper than that obtained using a slow sweep (*t*_int_ = 30 s). Although the output power obtained from a fast sweep may appear larger, this can be misleading. This is because the TEG cannot reach a thermal steady state in the case of a fast sweep (*t*_int_ = 0 s), owing to the slow thermal response. This explains why the TEG exhibited hysteresis in its *I–V* measurements during the fast sweep (*t*_int_ = 0 s). Therefore, the accuracy of the *I–V* measurements performed for a TEG depends on realization of a thermal steady state, which is best achieved with a sufficiently slow sweep speed (*t*_int_ = 30 s in our case). Again, Our results suggest that, for accurate measurement of the *I–V* characteristics of a 2T PV–TEG, the *t*_int_ between the measurement points should be carefully selected.

The impact of *R*_TEG_ should be minimized to obtain a loss-free PV–TEG system for field-scale applications. This can be achieved using one of two approaches: lowering *R*_TEG_ or incorporating a PV that is less susceptible to the effects of *R*_TEG_. Although *R*_TEG_ can be easily reduced in practice by decreasing the number of series connections and increasing the number of parallel connections in the TEG array, this is not desirable because it increases the operational current and heightens the risk of Joule heating proportional to *I^2^R.* Furthermore, because a TEG array with parallel connections provides a low TE electromotive force, this configuration does not yield an efficient PV–TEG. The second method for reducing the *R*_TEG_ is to construct a TEG array from low-resistance TE elements. As regards the electron motion across the TEG, the Peltier coefficient *Π* is expressed as

$\Pi=\frac{1}{q}\left[ \left( E_{c}-E_{Fn} \right)+\frac{3}{2}kT \right]$	(S1)

where *q* is the elementary charge, *k* is the Boltzmann constant, and *E*_c_ – *E*_Fn_ is the energy separation of the conduction band (*E*_c_) from the Fermi level (*E*_Fn_), considering an *n*-type semiconductor [S1]. Eq. (S1) suggests that heat exchange depends on the energy separation from the *E*_F_. Therefore, a semiconductor with either a low doping concentration or a large bandgap yields a highly efficient heat exchange. In other words, although a TEG can be composed of low-resistance TE elements characterized by a highly doped, small-bandgap semiconductor, this material may not function as a highly efficient TEG because the TEG heat exchange depends on the energy separation from *E*_Fn_, that is, the energy barrier. This implies that a TEG component composed of low-resistance TE elements operates at low voltage, which would be an ineffective contributor to PV–TEG. This is because TEG should add sufficient TE electromotive force (*V*_TEG_) to the PV–TEG. Furthermore, as discussed above, a low-resistance TEG would cause the PV–TEG to operate at higher current levels, thereby generating an even greater risk of Joule heating, which is proportional to *I^2^R*. Therefore, an effective TEG should have sufficient resistance to generate a high *V*_TEG_. However, an excessively high *R*_TEG_ would more significantly affect the PV–TEG and seriously decrease the fill factor (*FF*), as shown in Supplementary Fig. S6.

**Supplementary Note S2: Numerical model.**

We established a numerical model for the PV–TEG using a modified double diode model (MDDM). The *I–V* curve for this two-terminal (2T) PV–TEG can be described as follows:

$I =I_{PV}-I_{D1}-I_{D2}-\frac{V-V_{TEG}+I\left( R_{PV}+R_{TEG} \right)}{R_{\mathrm{sh}}}$	(S2)

where *I* is the terminal current of the PV–TEG; *V* is the applied voltage; *V*_TEG_ is the electromotive force from the TEG; $I_{\mathrm{PV}}$ is the photocurrent generated by the solar cell; $I_{D1}$ and $I_{D2}$ represent the diffusion and recombination currents, respectively; *R*_PV_ is the series resistance of the solar cell; *R*_TEG_ is the TEG resistance; and *R*_sh_ is the parallel resistance [S7–S9].

Additionally, we define

$V_{\mathrm{TEG}}=N_{s}\times S\times\Delta T$	(S3)

$R_{\mathrm{TEG}}=\frac{N_{s}R_{i}}{N_{p}}$	(S4)

where *N*_s_ is the number of TE elements in series, *S* is the Seebeck coefficient, Δ*T* is the TEG temperature gradient, *N*_p_ is the number of TE elements in parallel, and *R*_i_ is the internal resistance of the TE elements [S7]. By applying the TEG parameters, we can express *I*_D1_ and *I*_D2_ as follows:

$I_{D1}=I_{01}\left[ \exp\left( \frac{V-V_{\mathrm{TEG}}+I\left( R_{\mathrm{PV}}+R_{\mathrm{TEG}} \right)}{Nn_{1}V_{\mathrm{th}}} \right)-1 \right]$	(S5)

$I_{D2}=I_{02}\left[ \exp\left( \frac{V-V_{\mathrm{TEG}}+I\left( R_{\mathrm{PV}}+R_{\mathrm{TEG}} \right)}{Nn_{2}V_{\mathrm{th}}} \right)-1 \right]$	(S6)

where *I*_01_ and *I*_02_ are the recombination currents; *N* is the number of strips in the shingled module; and *n*_1_ and *n*_2_ are ideality factors. In Addition, *V*_th_ is the thermal voltage defined by the Boltzmann constant *k*, operating temperature *T*, and electron charge *q*, and is expressed as follows:

$V_{\mathrm{th}}=\frac{kT}{q}$	(S7)

For the two exponential functions in Eqs. (S5) and (S6) to be converted to the Lambert *W* function, they must be combined into a single exponential function. Therefore, the following steps are performed:

$\exp\left( \frac{V-V_{\mathrm{TEG}}+I\left( R_{\mathrm{PV}}+R_{\mathrm{TEG}} \right)}{NV_{\mathrm{th}}} \right)=\left( \frac{I_{D1}+I_{01}}{I_{01}} \right)^{n_{1}}=\left( \frac{I_{D1}+I_{02}}{I_{02}} \right)^{n_{2}}$	(S8)

We consider the following relationships:

$I_{D1}+I_{01}=\frac{I_{01}}{I_{02}^{n_{2}/n_{1}}}{(I_{D2}+I_{02})}^{n_{2}/n_{1}}$	(S9)

As $I_{01}$ and $I_{02}$ are smaller than $I_{D1}$ and $I_{D2}$, they can be neglected. By setting *a* = $\frac{I_{01}}{I_{02}^{n_{2}/n_{1}}}$ and *b* = $n_{2}/n_{1}$, **Eq. (S9)** can be rewritten as follows:

$I_{D1}=aI_{D2}^{b}$	(S10)

The exponential terms in the MDDM can now be separated as follows: For convenience, we simplify **Eq. (S10)** to the following linear equation:

$I_{D1}=\kappa I_{D2}+\tau$	(S11)

We then calculated various electrical characteristics at different irradiances and temperatures using the following equations (S12)–(S15). We obtained three different sets of diode currents, $\left( I_{D1\_SC}, I_{D2\_SC} \right)$,$\left( I_{D1\_OC}, I_{D2\_OC} \right)$, and $\left( I_{D1\_mp}, I_{D2\_mp} \right)$. These sets were fitted using the least-squares method to determine the coefficients *κ* and *τ*.

$I_{\mathrm{SC}}=I_{SC\_STC}\frac{G}{G_{\mathrm{STC}}}[1+K_{I}(T-T_{\mathrm{STC}})]$	(S12)

$V_{\mathrm{OC}}=V_{OC\_STC}+V_{\mathrm{th}}\ln\left( \frac{G}{G_{\mathrm{STC}}} \right)+K_{V}(T-T_{\mathrm{STC}})$	(S13)

$I_{\mathrm{mp}}=I_{mp\_STC}\frac{G}{G_{\mathrm{STC}}}[1+K_{\mathrm{IP}}(T-T_{\mathrm{STC}})]$	(S14)

$V_{\mathrm{mp}}=V_{mp\_STC}+V_{\mathrm{th}}\ln\left( \frac{G}{G_{\mathrm{STC}}} \right)+K_{VP}(T-T_{\mathrm{STC}})$	(S15)

where *G* is the solar irradiance, *T* is the temperature, *I*_mp_ is the current at the maximum power point, and *V*_mp_ is the voltage at the maximum power point. In Addition, *K*_I_, *K*_V_, *K*_IP_, and *K*_VP_ are the temperature coefficients of the short-circuit current (*I*_sc_), the open-circuit voltage (*V*_oc_), *I*_mp_, and *V*_mp_, respectively. The subscript STC refers to the solar-cell standard test conditions (STC); that is, 25 °C, AM1.5G, and 1 sun (100 mW/cm^2^).

Eq. (S2) can be split into Eqs. (S16) and (S17) by substituting Eq. (S11) into the MDDM. Equations comprising an exponential term can be expressed using the Lambert *W* function as follows:

$I=I_{\mathrm{PV}}-\frac{\kappa+1}{\kappa}\cdot I_{01}\left[ \exp\left( \frac{\left( V-V_{\mathrm{TEG}} \right)+I\left( R_{\mathrm{PV}}+R_{\mathrm{TEG}} \right)}{Nn_{1}V_{\mathrm{th}}} \right)-1 \right]+\frac{\tau}{\kappa}-\frac{\left( V-V_{\mathrm{TEG}} \right)+I\left( R_{\mathrm{PV}}+R_{\mathrm{TEG}} \right)}{R_{\mathrm{sh}}}$	(S16)

$I=I_{\mathrm{PV}}-\kappa+1\cdot I_{02}\left[ \exp\left( \frac{\left( V-V_{\mathrm{TEG}} \right)+I\left( R_{\mathrm{PV}}+R_{\mathrm{TEG}} \right)}{Nn_{2}V_{\mathrm{th}}} \right)-1 \right]-\tau-\frac{\left( V-V_{\mathrm{TEG}} \right)+I\left( R_{\mathrm{PV}}+R_{\mathrm{TEG}} \right)}{R_{\mathrm{sh}}}$ (S17)

Now, the current can be expressed using the Lambert *W* function as follows:

$I=\frac{R_{\mathrm{sh}}\left( I_{\mathrm{PV}}+\frac{\tau}{\kappa}+\frac{\kappa+1}{\kappa}\cdot I_{01} \right)-(V-V_{\mathrm{TEG}})}{R_{\mathrm{PV}}+R_{\mathrm{TEG}}+R_{p}}-\frac{{Nn}_{1}V_{\mathrm{th}}}{R_{\mathrm{PV}}+R_{\mathrm{TEG}}}W(x_{1})$	(S18)

$I=\frac{R_{\mathrm{sh}}\left( I_{\mathrm{PV}}-\tau+(\kappa+1)\cdot I_{02} \right)-(V-V_{\mathrm{TEG}})}{R_{\mathrm{PV}}+R_{\mathrm{TEG}}+R_{p}}-\frac{{Nn}_{2}V_{\mathrm{th}}}{R_{\mathrm{PV}}+R_{\mathrm{TEG}}}W(x_{2})$	(S19)

where

$x_{1}=\frac{\frac{\kappa+ 1}{\kappa}I_{01}(R_{\mathrm{PV}}+R_{\mathrm{TEG}})R_{\mathrm{sh}}}{Nn_{1}V_{\mathrm{th}}\left( R_{\mathrm{PV}}+R_{\mathrm{TEG}}+R_{\mathrm{sh}} \right)}\exp\left\{ \frac{R_{\mathrm{sh}}\left[ (R_{\mathrm{PV}}+R_{\mathrm{TEG}})\left( I_{\mathrm{PV}}+\frac{\tau}{\kappa} \right)+\frac{\kappa+1}{\kappa}\cdot I_{01}(R_{\mathrm{PV}}+R_{\mathrm{TEG}})+(V-V_{\mathrm{TEG}}) \right]}{Nn_{1}V_{\mathrm{th}}\left( R_{\mathrm{PV}}+R_{\mathrm{TEG}}\text{+}R_{\mathrm{sh}} \right)} \right\}$	(S20)

$x_{2}=\frac{(\kappa+ 1)I_{02}(R_{\mathrm{PV}}+R_{\mathrm{TEG}})R_{\mathrm{sh}}}{{Nn}_{2}V_{\mathrm{th}}(R_{\mathrm{PV}}+R_{\mathrm{TEG}}+R_{\mathrm{sh}})}\exp\left\{ \frac{R_{\mathrm{sh}}\left[ (R_{\mathrm{PV}}+R_{\mathrm{TEG}})\left( I_{\mathrm{PV}}-\tau\right)+(\kappa+1)\cdot I_{02}(R_{\mathrm{PV}}+R_{\mathrm{TEG}})+(V-V_{\mathrm{TEG}}) \right]}{Nn_{1}V_{\mathrm{th}}\left( R_{\mathrm{PV}}+R_{\mathrm{TEG}}\text{+}R_{\mathrm{sh}} \right)} \right\}$	(S21)

Dividing both sides of **Eq. (S18)** by $\frac{\kappa+1}{\kappa}$, and both sides of **Eq. (S19)** by κ + 1, our numerical model for the PV–TEG using MDDM based on the Lambert *W* function is as follows:

$I =\frac{R_{\mathrm{sh}}\left( I_{\mathrm{PV}}+I_{01}\text{+}I_{02} \right)-(V-V_{\mathrm{TEG}})}{\left( R_{\mathrm{PV}}+R_{\mathrm{TEG}}\text{+}R_{\mathrm{sh}} \right)}-\frac{{NV}_{\mathrm{th}}}{R_{\mathrm{PV}}+R_{\mathrm{TEG}}}\left[ \frac{\kappa}{\kappa+1}n_{1} W{(x}_{1})+\frac{1}{\kappa+1}n_{2} W\left( x_{2} \right) \right]$	(S22)

This model employs the Lambert *W* function to calculate the current. Therefore, we simulated *I–V* curves and compared them with the measured *I–V* curves to obtain the maximum coefficient of determination, as shown in Supplementary Fig. S7. Loss analysis typically involves fitting the measured *I–V* curve to a numerical model that describes the modeled *I–V* curves of the PV–TEG. Here, the fitting parameters of the numerical model were *I*_01_, *I*_02_, *R*_PV_, *R*_TEG_, and *R*_sh_. By adjusting these parameters during the fitting process, the model was optimized to match the measured data. For example, the *R*_TEG_ of the PV–TEG could be deduced from the numerical model. Notably, applying our numerical model to the measured *I–V* curves of the PV–TEG revealed a best fitting for *R*_TEG_ at 1.22 Ω instead of the nominal value of 0.88 Ω. This modeling outcome implies an increase in *R*_TEG_ resulting from Joule heating. Once the proposed model is well fitted to the experimental data, the PV–TEG parameters, such as the maximum output power (*P*_max_), *I*_mp_, *V*_mp_, *I*_sc_, *V*_oc_, and *FF*, can be determined.

For the loss analysis, the *P*_max_ of the 2T PV–TEG (*P*_max-2T_) was calculated by considering various combinations of *I*_mp_ and *V*_mp_. The calculated *P*_max-2T_ value was subsequently compared with the *P*_max_ of the four-terminal (4T) PV–TEG (*P*_max-4T_), as illustrated in Supplementary Fig. S8. *P*_max-4T_ was derived by computing the output power of the PV-only case using the MDDM models [S8,S9] and adding it to the measured output power of TEG. Therefore, the power loss (*P*_loss_) can be expressed as follows:

$P_{\mathrm{loss}} (\%)=\left( \frac{P_{max-4T}-P_{max-2T}}{P_{max-4T}} \right)\times100$	(S25)

To model *P*_max-2T_, we employed *R*_TEG_ = 1.22 Ω and Δ*T* = 25 °C, whereas for *P*_max-4T_, we used *R*_TEG_ = 0.88 Ω and Δ*T* = 25 °C.

Supplementary references

[S1]	*S.O. Kasap, Principles of electronic materials and devices* 488–492 (McGraw-Hill, 2017).

[S2]	G.E. Shoemake, J.A. Rayne, R.W. Ure, Specific heat of *n*- and *p*-type Bi_2_Te_3_ from 1.4 to 90 °K, Phys. Rev. 185, 1046.

[S3]	H. Köhler, Non-parabolicity of the highest valence band of Bi_2_Te_3_ from Shubnikov-de Haas effect. *Phys. Status Solidi, B* **74**, 591–600 (1976).

[S4]	Bhosle, V., Tiwari, A. & Narayan, J. Metallic conductivity and metal-semiconductor transition in Ga-doped ZnO. *Appl. Phys. Lett.* **88**, 032106 (2006).

[S5]	Kasap, S. O. *Principles of electronic materials and devices* (McGraw Hill, New York, 2017).

[S6]	Min, G. New formulation of the theory of thermoelectric generators operating under constant heat flux. *Energy Environ. Sci*. **15**, 356–367 (2022).

[S7]	Park, K.-T. et al. Lossless hybridization between photovoltaic and thermoelectric devices. *Sci. Rep.* **3**, 2123 (2013).

[S8]	Chen, Y., Sun, Y. & Meng, Z. An improved explicit double-diode model of solar cells: Fitness verification and parameter extraction. *Energy Convers. Manag.* **169**, 345–358 (2018).

[S9]	Jain, A. Exact analytical solutions of the parameters of real solar cells using Lambert *W*-function. *Sol. Energy Mater. Sol. Cells* **81**, 269–277 (2004).
